# Surface Plasmon Enhanced Photoluminescence of Carbon Dots Formed In Situ on Silver Gratings

**DOI:** 10.1002/advs.202523200

**Published:** 2026-03-09

**Authors:** Maryam Sadat Amiri Naeini, Jaspreet Walia, Luis Angel Mayoral‐Astorga, Hyung Woo Choi, Arnaud Weck, Pierre Berini

**Affiliations:** ^1^ Department of Physics University of Ottawa Ottawa Ontario Canada; ^2^ Nexus for Quantum Technologies Institute University of Ottawa Ottawa Ontario Canada; ^3^ School of Electrical Engineering and Computer Science University of Ottawa Ottawa Ontario Canada; ^4^ Department of Mechanical Engineering University of Ottawa Ottawa Ontario Canada

**Keywords:** carbon dot, fluorescence lifetime, grating, plasmon‐enhanced fluorescence, SERS, silver, surface plasmon

## Abstract

We report the enhanced photoluminescence of carbon dots formed in situ on silver gratings via plasmonic reduction of a carbonaceous seed layer. This layer transforms into photoluminescent carbon dots in a non‐oxygenated environment, catalyzed by surface plasmon polaritons (SPPs) excited on the grating by a pump laser, as monitored in real‐time via Raman scattering. Pump SPPs subsequently excite the carbon dots, which emit at longer wavelengths into the local electromagnetic environment, which is dominated by SPPs propagating on and diffracting from the grating. We experimentally observe SPP‐enhanced photoluminescence as a significant reduction in the lifetime of the emitters, and as induced coherence in far‐field emission. Purcell factors of ∼70 are deduced from lifetimes of ∼30 ps for carbon dots measured using time‐correlated single‐photon counting. SPPs accelerate radiative decay and produce polarized directional free‐space emission via grating diffraction, conferring characteristics of coherence to a random arrangement of luminescent carbon dots. A method for modeling an ensemble of random, incoherent emitters using finite‐difference time‐domain (FDTD) simulations is proposed, and the results agree with observed far‐field images and measured Purcell factors. Our results provide insight into carbon dot ‐ SPP interactions and have implications for cavity‐enhanced biosensors, quantum emitters, and carbon‐based light sources.

## Graphical abstract

Carbon emitters formed in situ on a silver grating via plasmonic catalysis yield enhanced spontaneous emission by preferential radiative decay into surface plasmons. Enhancement manifests as emission exhibiting characteristics of coherence (polarization, directionality), and accelerated lifetimes of ∼30 ps, yielding Purcell factors of ∼70. Findings have implications for cavity‐enhanced biosensors, quantum light sources, and carbon‐based light emitters.

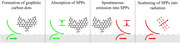

## 1 Introduction

It was initially explained by Purcell that the lifetime of an excited fluorophore is greatly affected by the electromagnetic environment in which the emitter resides [1]. In fact, modifying the local density of optical states (LDOS) near the emitter results in modification of its total decay rate to lower energy states, and accordingly changes its spontaneous emission rate relative to that in free space, by controlling the emission channels into which quanta decay [2, 3]. It is then expected that quantum emitters placed on a metallic surface that supports surface plasmon polariton (SPP) modes will interact strongly with the SPPs through absorption and emission processes. The former leads to efficient pumping, and the latter increases the fluorophore decay rate and the quantum yield, and introduces emission directionality, resulting in a modified radiation pattern compared to the fluorophore in free space [2, 4, 5, 6].

SPPs are surface waves comprising collective oscillations of electrons coupled to electromagnetic fields. SPPs are known to be highly confined and to have enhanced electromagnetic fields at specific locations on the surface of metallic structures – often termed hot spots. In addition to enhancing the emission rate of fluorophores via increased LDOS and field enhancement [7], these hot spots also provide an opportunity to excite (pump) fluorophores at a higher rate compared to free space. This increase is proportional to the square of the electric field enhancement factor [8]. Furthermore, plasmonic structures can be designed to compensate for the wavevector mismatch between SPP modes on the surface and radiative emission channels into free space. As a result, once fluorescence emission couples to SPP modes, the structure can be engineered to outcouple the SPP modes into desirable free space radiation channels. Modifying the radiation pattern of fluorophores is thus the third important role that a plasmonic nanostructure placed in proximity to a quantum emitter may play [9], after enhancing the emission rate [7] and the pump efficiency [8].

Plasmonic enhanced fluorescence and light‐emitting nanostructures have been the subjects of extensive research, culminating in several review papers (e.g., [2, 6, 10, 11]). For example, it has been shown that plasmonic grating structures enhance the spontaneous emission of fluorophores [12], and for emitters that have an inherently small radiative efficiency, this can be beneficial for applications such as sensing [13]. Fluorescence enhancement by nanoantennas and their prospective application to high‐speed LEDs are discussed in [14]. Greffet et al. introduced a new formulation as the local form of Kirchhoff's law to design and analyze thermal emitters and complex photo‐ or electro‐luminescent systems [15, 16], building on earlier work reporting a coherent thermal light source emitting a polarized, highly directive, and spectrally narrow beam [17]. A silver 2D metasurface hosting semiconductor nanocrystals and emitting in a narrow spectral range with high directivity was also designed using this method [9]. Knoll et al. measured the enhanced fluorescence emission of dye molecules coupled via SPPs to diffraction orders of a gold plasmonic grating [18, 19]. In a different study, they reported the spatial correlation between the excitation and emission of dye molecules close to a grating surface, due to localized plasmonic fields [20]. Barnes et al. explored dye molecule emission enhancement on plasmonic gratings and concluded that there are optimum grating ridge height and dye layer thickness for enhancing the emission intensity [21]. Also, they compared shallow (weakly perturbative) grating structures with a metallic mirror, revealing that the lifetime of *Eu* ^3 +^ ions does not differ significantly on these two types of structure [22].

Thus, plasmonic gratings are great candidates to enhance the pumping of fluorophores, their emission rate, their quantum efficiency, and to control the emission channels for polarization and directionality. A plasmonic grating has a broad resonance spectrum, and the grating pitch can be engineered so that its resonance covers both the absorption and emission wavelengths of emitters that have a small Stokes shift [23]. Alternatively, grating structures can also be designed to have two resonances, one overlapping with the absorption wavelength and the other with the emission wavelength of a species [24]. The grating wavenumber provides momentum mismatch compensation between free space photons and SPPs, beneficial for plasmon‐assisted pumping and fluorescence emission outcoupling to desired free space radiative channels [25]. SPP modes on a grating also create strongly confined electromagnetic fields over the illuminated portion of the metallic surface hosting the fluorescent species.

In previous work, we reported the plasmon‐catalyzed formation of photoluminescent carbon dots on gratings [26, 27], and ruled out thermal effects in the process. In this paper, we report the theoretical and experimental investigation of plasmon‐mediated pumping, emission, and outcoupling into free‐space radiation of photoluminescence produced by carbon dots formed in situ via plasmonic catalysis from a carbonaceous seed layer on shallow silver gratings. We also determine the lifetime of the carbon dots on silver gratings from which we deduce the Purcell factor of the system and the corresponding free‐space lifetime of the carbon dots. We investigate the far field radiation patterns of the emission and observe coherence in the form of directional and polarized emission. The role of plasmons in our system is four‐fold – first, pump plasmons catalyze the seed layer into graphitic carbon dots; second, pump plasmons excite the carbon dots; third, surface plasmons receive Stokes‐shifted quanta of energy emitted by decay of excited carbon dots; and fourth, surface plasmons scatter from the grating into specific free‐space radiation channels. Surface plasmons therefore, mediate photoluminescence by the carbon dots, significantly altering their spontaneous emission lifetime and radiation characteristics into free space. The paper is organized following these distinct roles.

## 2 Results and Discussion

### 2.1 In Situ Formation of Carbon Dots Catalyzed by Surface Plasmons

**FIGURE 1:**
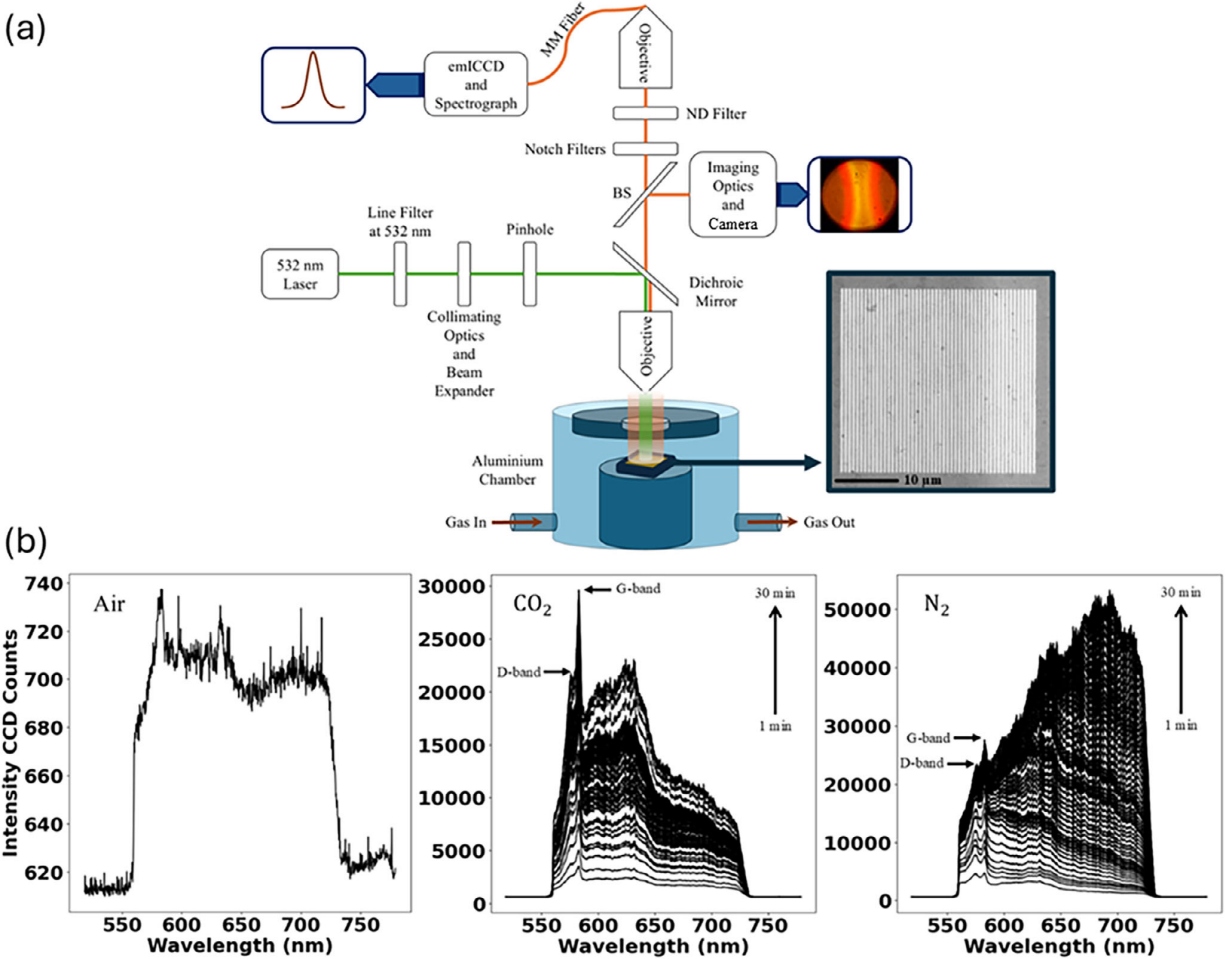
(a) Custom‐built Raman microscopy measurement setup using a CW laser emitting at λ = 532 nm. The setup includes the gas chamber containing grating structures and gas flow at ∼1.95 atm pressure. A spectrometer coupled to an emICCD camera is used to measure the spectra of the gratings in air and in both gases. The two insets show a camera image of a grating fluorescing in *N*_2_, and a HIM image of a grating structure post‐fabrication. (b) Initial SERS spectrum of the carbonaceous seed layer on the grating collected in air, and evolution of SERS spectra emanating from two gratings under 1.95 atm *CO*_2_ and *N*_2_ gases respectively. The structures are pumped at λ = 532 nm in all cases.

The structures consist of silver gratings of pitch Λ = 500 nm and ridge height d = 25 nm with a 50% duty cycle, formed by helium‐beam lithography, evaporation, and lift‐off (as detailed under “Methods”), on a 100 nm thick silver layer on a silicon substrate. The gratings were designed for efficient coupling to a normally‐incident *p*‐polarized loosely focused beam at a free‐space wavelength of λ = 532 nm. Each grating has an area of 30 µm × 30 µm. The inset in Figure 1a shows a helium ion microscope (HIM) image of a grating structure.

Post‐fabrication, the gratings bear carbonaceous residue left behind by the resist after lift‐off, which acts as a seed layer for the formation of graphitic carbon dots [27]. The gratings were inserted in a gas chamber with a gas flow of CO_2_ or N_2_ at ∼1.95 atm pressure, and a grating was excited with a *p*‐polarized 10 mW continuous wave (CW) laser beam at λ = 532 nm in the oxygen‐depleted environment to transform the carbonaceous seed layer into light‐emitting carbon dots. Surface enhanced Raman scattering (SERS) spectra were acquired simultaneously using a custom‐built setup, as sketched in Figure 1a and detailed under “Methods.”

Figure 1b shows the SERS spectra collected from a grating of pitch Λ = 500 nm, under ∼1.95 atm CO_2_ and N_2_ gases, over 30 min of illumination in the setup shown in Figure 1a. Individual spectra were collected with an integration time of 1 s, then averaged over 30 spectra. Overall, 60 averaged SERS spectra are plotted, highlighting the evolution of the system over time as the grating is pumped. Spectra were observed to saturate and stabilize after about 30 min under illumination, consistent with previous work [27]. Graphene‐like peaks at λ = 572 nm (D‐band) and λ = 580 nm (G‐band) are observed to emerge from the carbonaceous seed layer on the structure. The peaks do not move, but their intensity grows over time, suggesting the continual transformation of the seed layer on the structure to organized carbon dots, via surface plasmon catalyzed transformation in an oxygen‐depleted environment [27]. These peaks are found precisely at Raman shifts of 1350 and 1580 cm^−1^, respectively, from the pump wavelength, corresponding to graphene‐like material. The broad Stokes‐shifted photoluminescence (PL) peaks at λ = 620 nm in CO_2_ and λ = 690 nm in N_2_ grow over 30 min, then remain stable afterward, while the laser power and gas pressure are maintained. The initial SERS spectrum of the carbonaceous seed layer in air is also plotted, showing weak PL, D‐, and G‐band peaks.

### 2.2 Pumping of Carbon Dots by Surface Plasmons

**FIGURE 2:**
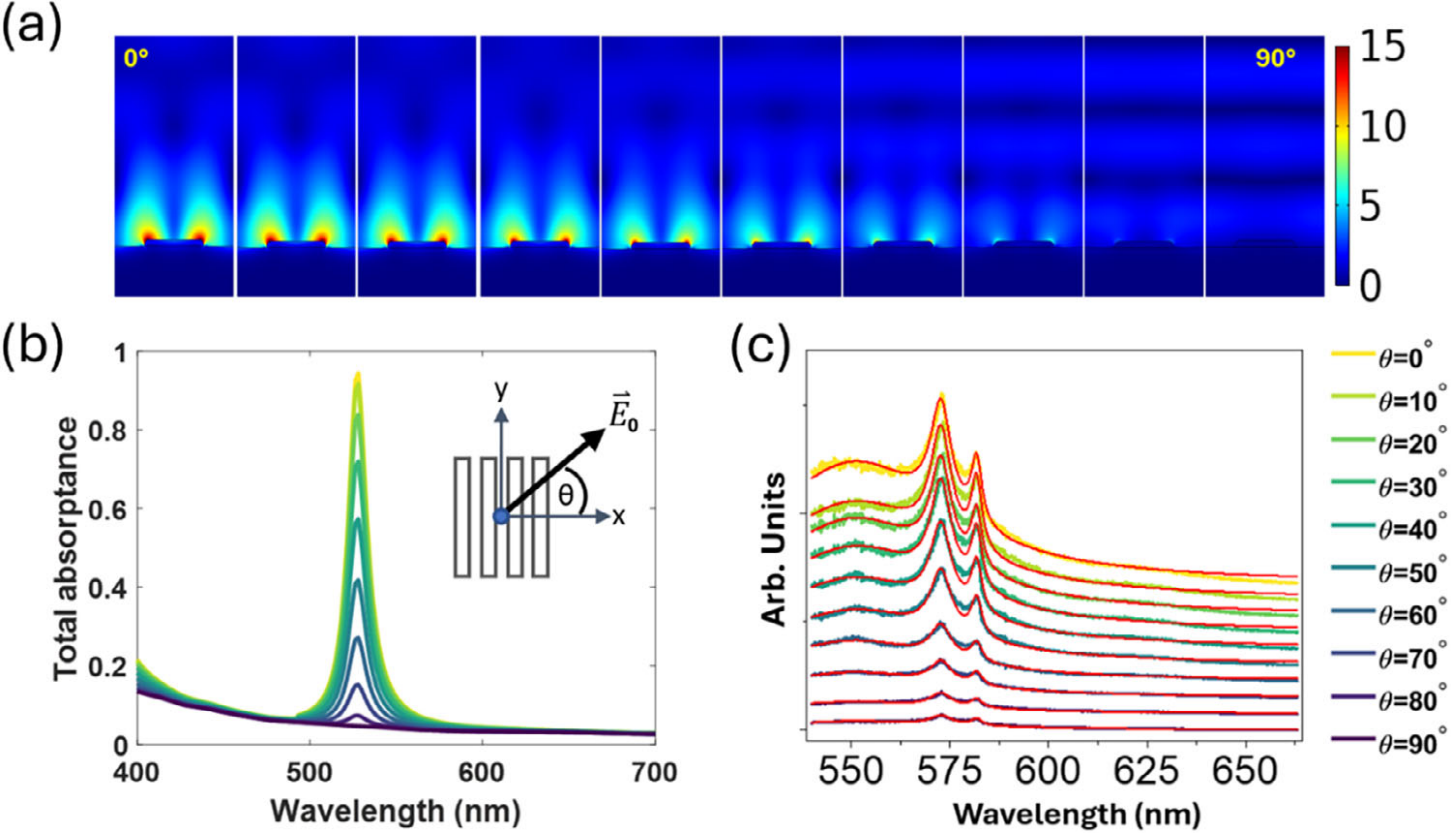
(a) Computed normalized total electric field distribution (*E*/*E*_0_) over a unit cell of an infinitely periodic silver grating of pitch Λ =  500 nm and height 25 nm, illuminated by a plane wave at λ = 532 nm (on resonance) and different polarization angles θ as noted on the panels and defined in the inset to Part (b). (b) Computed absorptance of an infinitely periodic grating illuminated by a plane wave at different polarization angles (yellow: θ = 0; dark purple: θ = 90°). (c) Measured SERS spectra of the structure in a gas chamber containing *CO*_2_, pumped at λ = 532 nm at different polarization angles. The solid curves are fitted to the measured data by decomposition of the spectra into a sum of Lorentzian functions (*cf*. Figure 3). The spectra are offset vertically for clarity. Parts (b) and (c) share the same color bar.

Figure 2a shows the electric field enhancement over a unit cell of an infinitely periodic silver grating, illuminated by a normally‐incident plane wave at λ = 532 nm (on resonance) for different polarization angles, computed using a commercial FEM analysis tool (as detailed under “Methods”). The grating ridge corners were rounded to a radius of 20 nm to avoid electric field divergence. Figure 2b shows the absorptance of the structure at the corresponding polarization angles, computed over the operating bandwidth of the grating. A (normally‐incident) *p*‐polarized (θ = 0) plane wave at λ = 532 nm couples most efficiently to SPPs on the grating, which leads to stronger local fields and greater absorptance. Thus, rotating the polarization provides a means for controlling the strength of the plasmonic pump on the grating, and the degree to which carbon dots thereon are excited.

Once the photoluminescence emanating from a grating stabilized after the formation of carbon dots, SERS spectra were collected as a function of incident polarization angle, as shown in Figure 2c. (These spectra were collected using a commercial Raman microscope ‐ WiTec a300 ‐ operating at λ = 532 nm, and are observed to be consistent with those of Figure 1b using our custom setup.) The spectrum obtained under *p*‐polarization (θ = 0) is the strongest, from which D and G peaks are readily observed, as well as a strong broadband photoluminescent background. As the incident polarization is rotated, the strength of the spectra drops to become barely measurable for *s*‐polarization (θ = 90°) but remains otherwise qualitatively similar. This behavior is readily understood considering Figure 2a: under *s*‐polarization, coupling to SPPs is not possible, so the grating behaves essentially as a flat mirror along which the total fields are weak. Conversely, under *p*‐polarization, SPPs are efficiently excited, resulting in strong (enhanced) fields along the surface, thereby enabling far more efficient pumping of the carbon dots thereon. Thus, polarization rotation conveniently enables control over the strength of the emission by modulating the pump interaction with the system.

**FIGURE 3:**
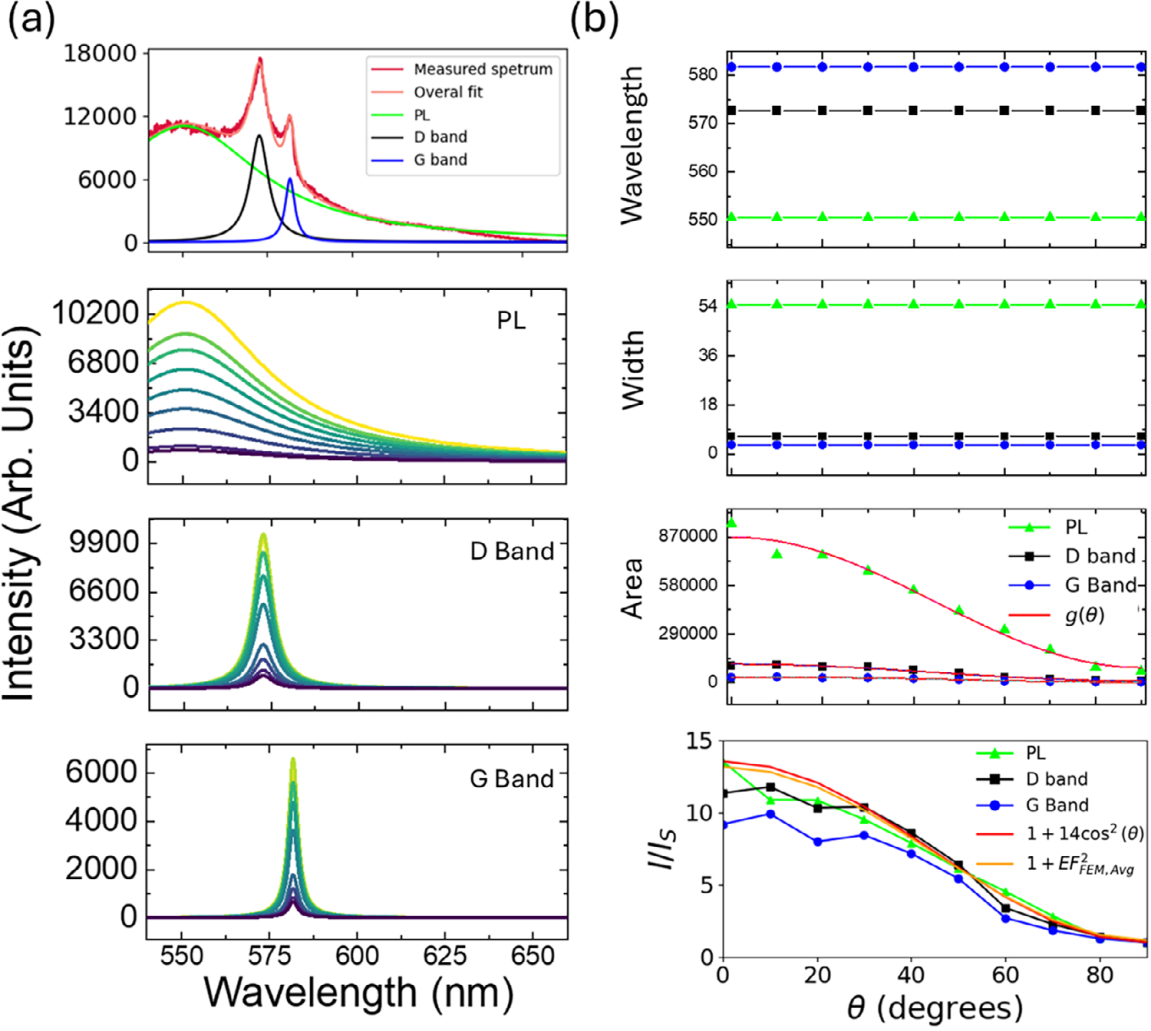
(a) Lorentzian decomposition extracted from the SERS spectra plotted in Figure 2c for different polarization angles. The top panel shows the decomposition and overall fit to experimental data for *p*‐polarization (θ = 0). (b) Peak parameters of the extracted Lorentzian functions of Part (a). The bottom two panels show the integrated intensity and the integrated intensity relative to *s*‐polarization (θ = 90*^o^*) of the peaks in panel (a) against polarization angle (θ). The integrated intensities follow Malus's law with an offset, g(θ) = Acos ^2^θ + B. The bottom panel of Part (b) also includes FEM simulation results in the form of the normalized squared mean field enhancement factor, ${\mathrm{EF}}_{\mathrm{FEM},\mathrm{Avg}}^{2}$ (*cf*. Figure 2a).

Decomposition of the measured spectra of Figure 2c into a sum of Lorentzian functions was performed, and the values of the Lorentzian peak position, linewidth, and integrated intensity were extracted for each excitation polarization. Results are summarized in Figure 3. Each spectrum fits very well to three Lorentzian functions, as shown for one case in Figure 3a, first (top) panel.

From Figure 3b, first and second panels, we observe that the center wavelength and width of the peaks remain unchanged at different polarization angles. The D‐band peak is centered at λ = 572 nm and the G‐band peak is centered at λ = 580 nm, as observed on other gratings in this study (*cf*. Figure 1b). However, the PL peak is centered at λ = 550 nm, which differs from the wavelength of the PL peak observed in Figure 1b for CO_2_. Indeed, it was observed through investigation of several structures over many fabrication iterations that the wavelength of the PL peak varies, likely due to variations in the formation of the carbon dots from one experiment to another.

The third panel of Figure 3b reveals that the integrated PL, D‐ and G‐band intensities, taken as the area under each Lorentzian, increase as the incident polarization becomes aligned with *p*‐polarization. The integrated intensities vary as a function of polarization angle similarly to Malus's law for the intensity of linearly polarized light passing through a polarizer [28]. Functions of the form g(θ) = Acos ^2^θ + B fit the integrated intensities with good agreement for the PL‐band (g(θ) = 7.8 × 10^5^cos ^2^θ + 9 × 10^4^), D‐band (g(θ) = 1 × 10^5^cos ^2^θ + 1 × 10^4^), and G‐band (g(θ) = 2.9 × 10^4^cos ^2^θ + 2.6 × 10^3^). The offset, B, in these expressions captures the weak intensity in the *s*‐polarized case.

Figure 3b bottom panel shows the integrated intensity of the three peaks normalized to their respective values in the *s*‐polarization case against polarization angle. Malus's law, scaled to the maximum experimental value with an offset, is also plotted for comparison (red curve). The computed distribution of the field enhancement factor shown in Figure 2a was sampled 3 nm above the silver surface and averaged over 15 points spanning the unit cell for each polarization angle, then normalized to the *s*‐polarization case, squared, and scaled to match the maximum experimental PL value, yielding the yellow curve which is nearly identical to the offset Malus law (red curve). All the measurement curves are observed to collapse approximately to this trend, which further corroborates our interpretation that the excitation of SPPs increases the strength of the SERS spectra, including the PL, due to the more efficient excitation of the carbon dots on the surface.

The localized enhanced electromagnetic fields of SPPs increase the absorption cross‐section of emitters as follows [8]:

(1) $$\sigma \left(x,y\right)={\left|EF\left(x,y\right)\right|}^{2}\sigma_{0}$$

with σ(*x*, *y*) and σ_0_ being the emitter's absorption cross‐section within the SPP electromagnetic fields and in free space, respectively, and EF(x, y) being the electric field enhancement factor of the SPP as a function of position (e.g., Figure 2a). This relationship is corroborated by the results presented in Figures 2c and 3. A *p*‐polarized beam illuminating the structure on resonance excites SPPs most effectively, leading to stronger localized fields, a greater field enhancement factor, more efficient pumping of the carbon dots, and consequently, brighter emission.

### 2.3 Decay of Carbon Dots into Surface Plasmons

#### 2.3.1 Theoretical

Once excited, the carbon dots emit into an electromagnetic environment that includes SPPs on the grating described by the LDOS on and near the grating. Specifically, SPPs on a grating increase the LDOS, which opens a quick radiative channel that competes better against nonradiative ones [7]. Following Equation (1) and the reciprocity theorem [29], it has been shown that the radiative decay rate of emitters coupled to SPPs (γ_r_) increases proportionally with the square of the electric field enhancement factor (EF) relative to the unperturbed (e.g., free space) emitter decay rate ($\gamma_{r}^{0}$) [30]:

(2) $$\gamma_{r}={\left|EF\right|}^{2}\gamma_{r}^{0}$$

As a result, the quantum yield (η) of such emitters depends on both their intrinsic and engineered radiative and nonradiative decay rates [5]:

(3) $$\eta =\frac{\gamma_{r}}{\gamma_{r}+\gamma_{nr}^{0}+\gamma_{\mathrm{LSW}}+\gamma_{\mathrm{abs}}}$$

where γ_r_ is the radiative decay rate of the emitter in its environment (near the grating in this case) and is related to the emitter's intrinsic radiative decay rate by the field enhancement factor following Equation (2). $\gamma_{\mathrm{nr}}^{0}$, γ_LSW_, and γ_abs_ are, respectively, the intrinsic nonradiative decay rate of the emitter, the dissipation rate into lossy and nonradiative electromagnetic modes that may be present on the structure surface (e.g., dark SPP resonant modes), and the absorption rate into the environment (including the metal). The denominator in Equation (3) is the total decay rate (γ) of the grating and emitter system:

(4) $$\gamma =\gamma_{r}+\gamma_{nr}^{0}+\gamma_{\mathrm{LSW}}+\gamma_{\mathrm{abs}}$$

and

(5) τ=1/γ

is the lifetime of the species in this system.

**FIGURE 4:**
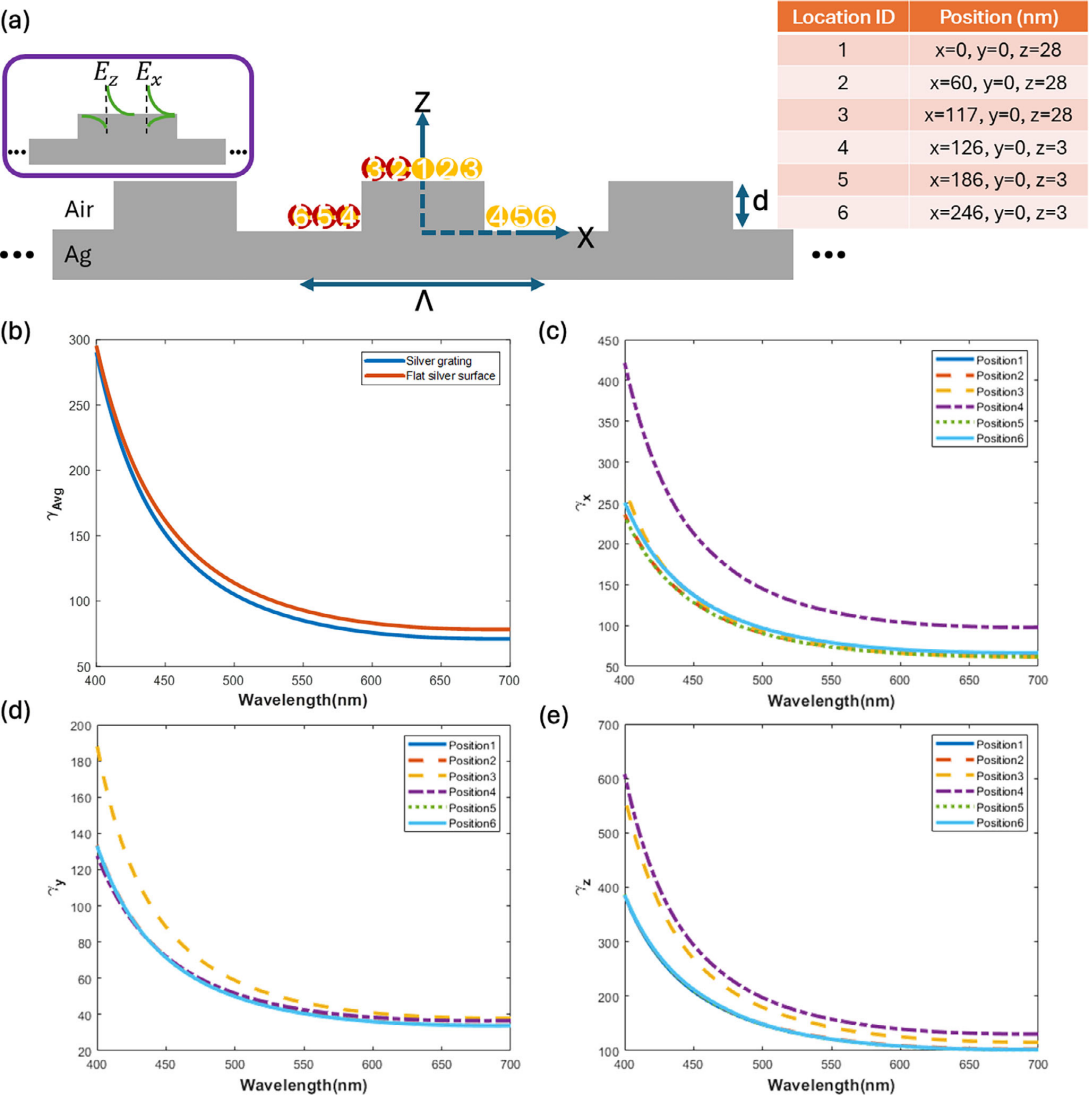
(a) Cross‐sectional schematic of a silver grating of pitch Λ = 500 nm, ridge height d = 25 nm, and extension along y of 2 µm, bearing dipoles, as modeled using the 3D FDTD method. The numbered orange balls identify the locations of point electric dipoles, which may be polarized along the x, y, or z directions (the red dashed contours are locations mirrored relative to the *z*‐axis). All dipoles were placed 3 nm above the metal surface. The inset on the left sketches the SPP mode fields for propagation along the *x‐axis*. (b) Normalized (dimensionless) decay rate averaged over all positions considered and over three dipole orientations (x, y, z), for the grating sketched in Part (a). Averaging for the case of the flat silver surface was taken only over dipole orientation, given the uniformity of the structure in the *x*‐*y* plane. (c–e) Normalized decay rate for dipoles located near the grating at positions 1 through 6, and oriented along the x, y, and z directions, respectively.

Figure 4 shows the total decay rate (Equation (4)) of point electric dipoles oriented and located in various ways over a finite grating, as a function of the oscillating wavelength, computed using the FDTD method. The intrinsic quantum efficiency of the dipole is assumed to be unity in these calculations, η_0_ = 1, i.e., we assume that nonradiative decay processes are not intrinsically present in the dipole ($\gamma_{\mathrm{nr}}^{0}=0$). The plotted decay rate is normalized to the decay rate of the dipole in free space (far from the grating). It is calculated by dividing the power dissipated by the dipole into the simulation environment by the power radiated by the dipole in free space (far from the structure) [31, 32, 33]. The total normalized decay rate is also termed the Purcell factor.

In each numerical calculation, a single dipole oscillating along the x, y, or z axes is placed at one of the locations identified by the orange balls in Figure 4a and the table inset into Figure 4a. The locations are chosen to sample the entire central unit cell of the grating while maintaining the required computational resources at a reasonable level. The average decay rate of emitters close to the grating structure is then found by averaging over the decay rates of all dipole orientations at positions sampling the entire central unit cell. This approach purports to model an ensemble of dipoles oriented randomly and located randomly over the grating surface. Further details on these computations and validation of our approach using the FDTD can be found under “Methods” and in Section S.1.

The calculated average decay rate of dipoles near a plane silver surface is also included for comparison, taken as the average over three simulations of single dipoles oscillating along the x, y, or z axes, but not over position, given the uniformity of the structure in the *x*‐*y* plane. It can be seen from Figure 4b that the average decay rate of emitters close to the plane silver surface is similar to the average decay rate close to the grating structure ‐ this is expected because the grating is shallow so the SPPs thereon are similar to those on the plane silver surface. In both systems, the decay rate increases as the wavelength decreases because SPPs become increasingly enhanced and localized to the interface. Thus, dipoles couple as efficiently to SPPs on a shallow metal grating as they do on a plane metal surface. However, importantly, the grating has diffraction orders that provide efficient outcoupling of SPPs to free space emission, as will be discussed further below.

The dipole oscillating along the *z‐axis* has the highest decay rate, as observed by comparing Figure 4c–e. The SPP mode is transverse magnetic (TM) (*p*‐polarized), with its main component of electric field normal to the metal/dielectric interface (E_z_). This causes the dipole oscillating along the z orientation (normal to the surface) to couple most efficiently to the SPP, resulting in a higher decay rate. SPPs excited by z‐oriented dipoles are launched isotropically (in all directions) in the *x*‐*y* plane of a shallow grating.

The SPP also possesses a longitudinal electric field (parallel to the propagation direction), which is generally in the *x*‐*y* plane. For example, SPPs that propagate in the x direction (perpendicular to the grating ridges) have an E_x_ field component, as sketched in the left inset to Figure 4a, and SPPs that propagate in the y‐direction (parallel to the grating ridges) have an E_y_ field component. Thus, dipoles that oscillate in the x or y directions also couple to SPPs, launching them in these specific directions. These dipole orientations exhibit a lower decay rate compared to z‐oriented dipoles (γ_x_, γ_y_ < γ_z_), as observed by comparing Figure 4c,d with Figure 4e, because the longitudinal field component of a SPP mode is weaker than the perpendicular one. The dipoles oscillating in other directions, e.g., at a 45° angle with respect to the *x‐axis*, are not considered in our simulations because all other in‐plane propagating modes can be decomposed into two orthogonally propagating modes along the x and y axes, and their effect is included by averaging.

Among all dipole positions considered, positions 3 and 4 correspond to locations where the dipole is near corners where SPP fields are more enhanced relative to other locations (*cf*. Figure 2a). As a result, the decay rate at these two locations is higher than at other locations. To prevent the sharpness of grating structure edges from affecting our results, the dipoles at positions 3 and 4 are located at a lateral distance of 3 and 6 nm from the edges, respectively. Also, since the perpendicular component of the electric field to the metal/dielectric interface rotates in these two locations (to stay normal to the grating ridge walls), γ_x_ at position 4 and γ_y_ at position 3 become larger than in other cases.

Equation (3) gives the quantum yield of an emitter into SPPs supported by the grating, but it does not include efficiency terms for diffraction into various orders, which could be calculated to assess the efficiency of a particular channel. Nonetheless, emission into free space due to outcoupling of SPPs by the grating can be detected, and measuring the lifetime of the emitted intensity can provide insight on the emission channels of the carbon dots formed on the grating, as discussed in the next section.

#### 2.3.2 Experimental

If the sample incorporates a single type of carbon dot, then the excitation and decay properties are identical over the ensemble, so the excited state population at each moment “t” after an excitation pulse depends only on the initial population of excited species, resulting in a single exponential intensity decay against time [7]:

(6) $$I\left(t\right)=I_{0}\exp\left(-\frac{t}{\tau }\right)$$

where *I*_0_ is the initial intensity (at *t* = 0) and τ is the lifetime of the emitters following Equation (5). When using the time correlated single photon counting (TCSPC) method, the measured decay curve of the emission intensity, *R*(*t*), is a convolution of the sample and measurement system time responses:

(7) $$R\left(t\right)=P\left(t\right)⊗I\left(t\right)$$

where *I*(*t*) is the sample response time and *P*(*t*) is the impulse or instrument response function (IRF) of the TCSPC measurement system. The latter is a convolution of the temporal response of all components involved in the measurement method, such as the laser pulse, detector diffusion, and transit time spread (TTS), electronic jitter, and dispersion in the optical components [7]. Convolution is required in Equation (7) because the excitation pulse and the detector's response are not immediate, and both have some temporal duration or reaction time. As a result, to extract the decay curve of emitters, I(t), one needs to deconvolve the impulse response function of the system from the measured histogram or decay curve. Such calculations are most critical for lifetime values comparable to or shorter than the IRF temporal width, and result in a more accurate decay curve including only the response of emitters. The slope of the decay curve plotted on a semilog scale, can be used to extract the lifetime of the sample with minimal errors.

**FIGURE 5:**
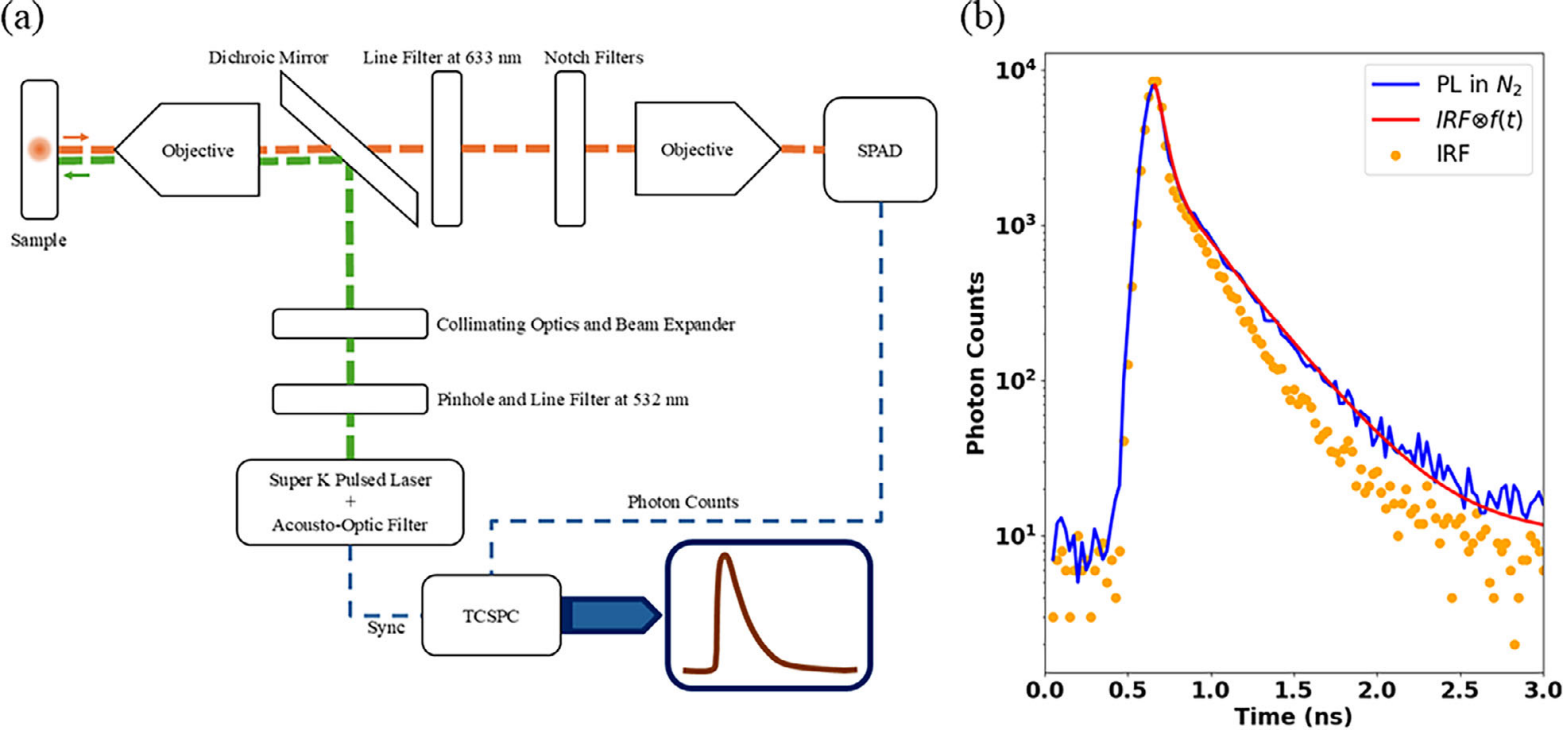
(a) Optical setup for the emission lifetime measurement of carbon dots on a silver grating. (b) IRF measured for the setup of Part (a) (orange dots) on a silver surface, and the lifetime measurement of the emission intensity emanating from a grating in *N*_2_ (blue curve). A theoretical fit extracting an exponential function of the form $f\left(t\right)=Ae^{-t/\tau }+Be^{-t/\tau_{SPAD}}+C$ is also plotted (red curve), where τ and τ_*SPAD*_ are the lifetime of the emitters and the decay time of carrier diffusion in the SPAD, respectively.

The optical setup used to measure the emitter lifetime is shown in Figure 5a. The setup is based on a pulsed supercontinuum source, optical filters of central wavelength λ = 532 nm, and a single photon avalanche diode (SPAD) connected to a TCSPC board. All decay curves were measured by capturing full emission spectra, except for one case corresponding to the emission lifetime at λ = 633 nm which was measured by adding a bandpass (line) filter in the collection arm right before the notch filters to only pass the emission component at λ = 633 nm. Further details on this setup and on measurement of the system IRF are given under “Methods.”

Figure 5b shows the measured IRF (orange dots) and emission decay curve (blue) collected from a silver grating in ∼1.95 atm N_2_, prepared as discussed in Sub‐Section 2. This measured decay curve is taken as the time‐discretized form of R(t) in Equation (7). We cannot use the single exponential I(t) of Equation (6) to model the decay of the species because the latter combines with the decay of diffused carriers in the SPAD [34, 35]. Instead we adopt a sum of exponentials of the form $f\left(t\right)=Ae^{-t/\tau }+Be^{-t/\tau_{\mathrm{SPAD}}}+C$ where τ and τ_SPAD_ represent the lifetime of the carbon dots (Equation (5)) and the SPAD diffusion tail respectively. f(t) is then time‐discretized and used in the discretized form of Equation (7), treating A, B, C, τ and τ_SPAD_ as fitting parameters. The resulting curve is plotted in Figure 5b (red). The corresponding fitting parameters are reported in Table 1 along with the reduced Poisson weighted χ^2^ representing the goodness of fit.

**TABLE 1:** Fitting parameters of the function $f\left(t\right)=Ae^{-t/\tau }+Be^{-t/\tau_{SPAD}}+C$ extracted from the lifetime measurements of Figures 5b and 6. The lifetime of the carbon dots on silver gratings in different gas environments is τ. The reduced Poisson weighted χ^2^ values are also listed as goodness of fits.

Experiment	A	B	C	τ_SPAD_ (ps)	τ (ps)	χ^2^
Grating 1 in N_2_ (Figure 5b)	4 × 10^13^	1.4 × 10^4^	10	330	30	1.635
Grating 2 in CO_2_ (Figure 6b)	9 × 10^13^	5 × 10^2^	10	330	40	1.933
Grating 3 in N_2_ (Figure 6c)	2.5 × 10^16^	1.2 × 10^4^	200	330	35	1.890
Grating 3 in N_2_, emission at λ=633 nm, (Figure 6d)	2.5 × 10^18^	8 × 10^4^	200	330	30	1.389
Dried dye coated on a silver surface (Figure S2)	6.9 × 10^19^	3.8 × 10^5^	500	330	30	1.969
1 ppm R6G solution in Ethanol (Figure S2)	9.9 × 10^3^	—	10	—	3960	—

**FIGURE 6:**
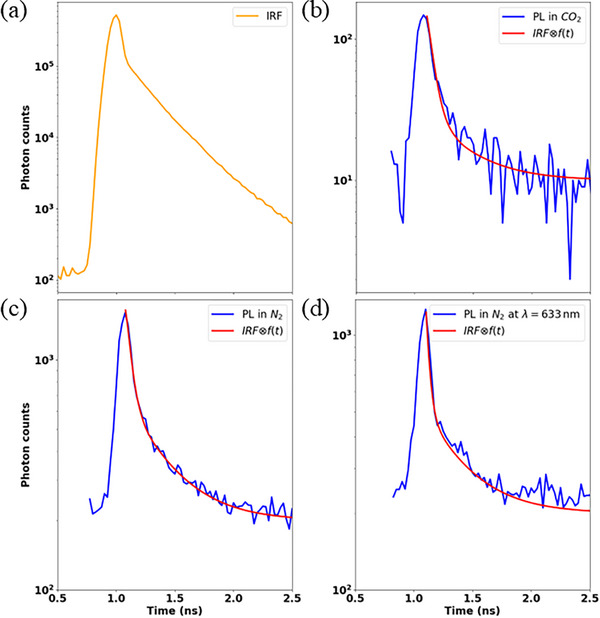
(a) IRF measured on a Si surface. Emission decay curves (blue) of the gratings characterized in Figure 1b in: (b) *CO*_2_, (c) *N*_2_, and (d) *N*_2_ through a line filter centered at λ = 633 *nm*. Theoretical fits extracting an exponential function of the form $f\left(t\right)=Ae^{-t/\tau }+Be^{-t/\tau_{SPAD}}+C$ are also plotted (red curves), where τ and τ_*SPAD*_ are the lifetime of the emitters and the decay time of carrier diffusion in the SPAD, respectively.

Figure 6a shows the IRF measured immediately prior to obtaining the results plotted in Figure 6b–d. Figure 6b–d plots the PL decay of the two gratings of Figure 1b in ∼1.95 atm CO_2_ and N_2_, collected during the same experimental run but after 30 min of CW beam exposure to grow and stabilize the PL spectra (as described in Sub‐Section 2.1). The PL in these cases was directly focused onto the SPAD in a free‐space arrangement to achieve the fastest IRF possible (Figure 6a), resulting in our highest fidelity lifetime measurements. The curves f(t) fitted to these measurements follow the same form as in Figure 5b, and the fitting parameters with χ^2^ are reported in Table 1 for each case.

The fitting results compiled in Table 1 show lifetimes of τ = 30 − 40 ps for carbon dots on silver gratings in different gases (holding the decay time for carrier diffusion in the SPAD constant at τ_SPAD_ = 330 ps in all cases. These lifetimes are shorter than the temporal width of the IRF (∼ 100 ps) and confirm the need for deconvolution of the IRF from experimental decay curves to achieve accurate results (*cf*. Equation (7)). Our lifetimes for carbon dots on a silver grating are much smaller than the reported lifetimes for carbon dots in a dielectric, which are in the range of 3–20 ns [36, 37]. Forming the ratio of our measured lifetimes with such values yields Purcell factors in agreement with those predicted in Figure 4, specifically, the averaged value in Figure 4b of $\gamma_{\mathrm{Avg}}∼80$ at λ∼600nm. Conversely, taking τ = 30 − 40 ps as the lifetime of our carbon dots on the silver grating, with γ_Avg_ =  80, yields $\tau_{0}∼2-3\;\mathrm{ns}$ for the lifetime of our dots in free space, in agreement with the range found in the literature.

These results were corroborated via reference measurements using R6G dye as described in Section S.2. Briefly, the lifetimes of R6G in solution and as a dried layer on a silver surface were measured, yielding a ratio (Purcell factor) of about 70, in good agreement with the predicted value in Figure 4b of $\gamma_{\mathrm{Avg}}∼80$ at λ∼600nm for a flat silver surface.

### 2.4 Surface Plasmon Scattering into Free Space Channels

The main difference between the behavior of emitters close to a plane surface vs. a grating is that the latter produces far field radiated fields, whereas the former, in the ideal case, does not (roughness could weakly outcouple SPPs into normally isotropic radiation). The SPPs excited on the grating by the emitters are efficiently outcoupled into various free‐space diffraction orders following energy and momentum conservation, with the grating compensating for any momentum mismatch between the SPPs and the radiated orders.

The momentum conservation equation relating the SPPs propagating on the grating in the x‐direction to the diffraction orders is written:

(8) $$\frac{\omega }{c_{0}}\sin\theta \pm N\frac{2\pi }{\Lambda }=\pm k_{\mathrm{SPP}}=\pm \frac{\omega }{c_{0}}\sqrt{\frac{\varepsilon_{d}\varepsilon_{m}}{\varepsilon_{d}+\varepsilon_{m}}}$$

where Λ is the grating pitch, *N* is the order, and θ and ω are the emission beam angle and frequency, respectively. *k*_SPP_ is the surface plasmon wavenumber in the x‐direction on the grating, and ε_d_ and ε_m_ are the relative permittivity of air and silver, respectively.

**FIGURE 7:**
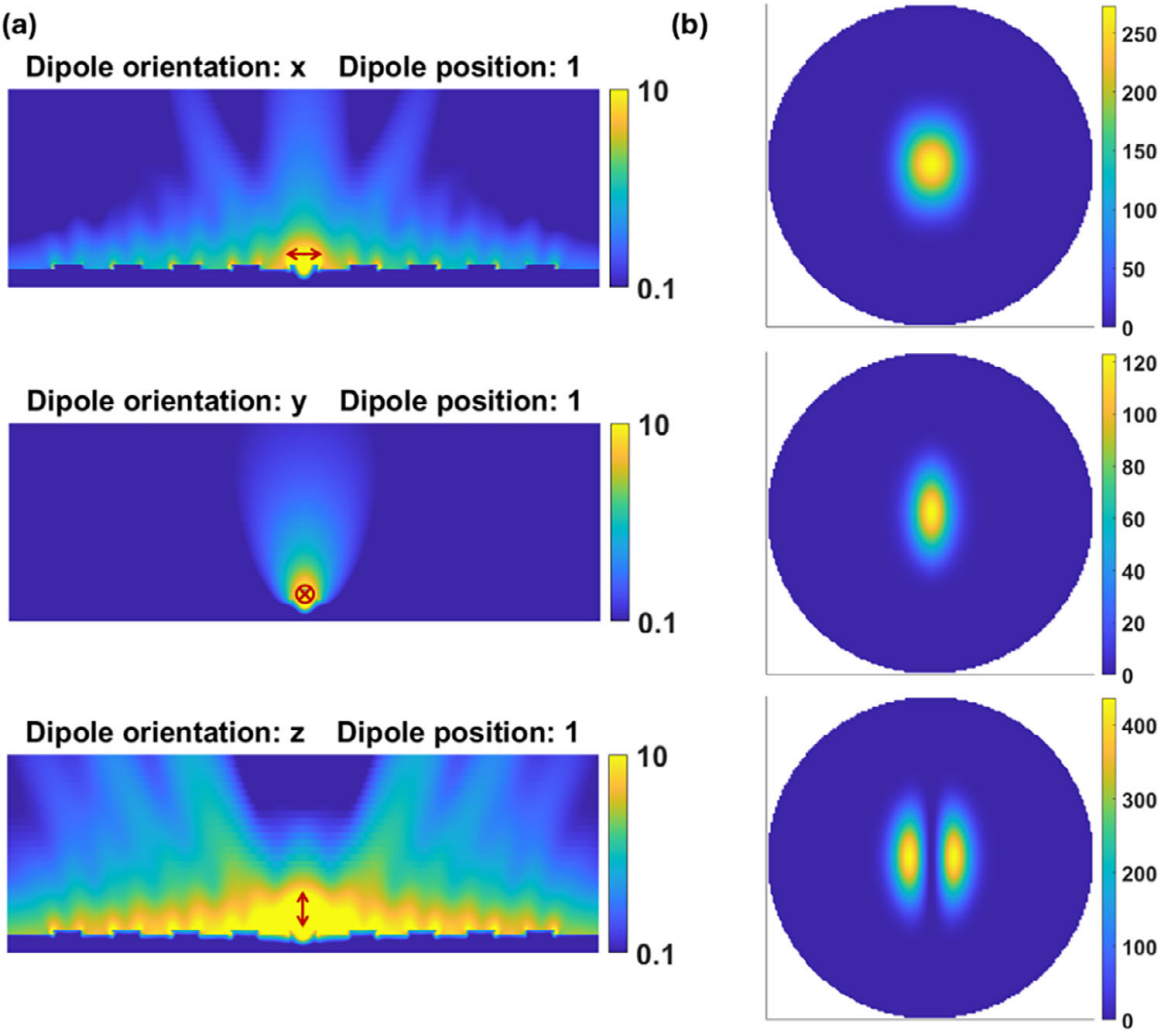
FDTD Simulations of a single electric dipole exciting a finite silver grating. (a) Near field plots of the magnitude of the total electric field |E_t_| for different dipole orientations placed at position 1 (*cf*. Figure 4a, table in inset). The color bar is in arbitrary units and saturated to better render the field distribution. (b) corresponding far field plots of |E_t_|^2^ normalized to the maximum squared magnitude of the far field radiated at the same location by an identical dipole oscillating in free space. The far field plots are produced from the fields at an *x*‐*y* plane 10 µm above the grating and projected on a 1 m radius sphere centered at the grating surface.

Figure 7a shows near‐field plots of the magnitude of the total electric field (|*E_t_* |) excited by a single dipole oscillating at λ = 560 nm and positioned above the center of the grating structure (position 1), for the 3 dipole orientations. The color bars in these cases are saturated in order to better render the near and radiated fields. Figure 7b shows the corresponding radiated far field squared magnitude (|*E_t_* |^2^). The far fields were computed by projecting the middle fields on the *x*‐*y* plane at a distance of 10 µm from the grating to a far field sphere of radius of one meter centered at the grating surface [38]. The far field plots are normalized to the maximum magnitude of the electric field produced at the same distance by a dipole oscillating in free space, and thus represent intensity enhancement or directivity.

As discussed earlier, it can be observed from these plots that the dipole oscillating normal to the surface (z‐orientation), excites the SPP modes more strongly and, as a result, can couple into free space emission channels more strongly as observed from the bottom panels of Figure 7. On the other hand, the dipole oscillating parallel to the grating ridges (y‐orientation) produces weaker coupling to SPP modes (*cf*. Figure 4d) and the weakest radiation, because the SPPs propagate along the y‐direction and are not outcoupled efficiently by the grating, as observed from the middle panels of Figure 7.

The far field plot for the dipole oscillating normal to the surface (z‐orientation), shows a central null with two lobes, as observed in Figure 7b, bottom panel. Recall that this dipole orientation launches SPPs propagating in phase and isotropically in the *x*‐*y* plane from the point of origin. The two emission lobes appear at specific angles in the *z*‐*x* plane relative to normal, determined by the first diffraction orders of the grating at the dipole wavelength. Substituting N = ±1 in Equation (8) yields emission angles of θ ≅ ±6^○^, which agree very well with the angles observed in the FDTD simulations of Figure 7, bottom panels. There is no emission in the normal direction because SPPs are in phase at the location of the dipole.

The field plots for the dipole oscillating in the x‐orientation reveal emission also along the first diffraction orders at the same angles, but additionally along the grating normal, as observed in Figure 7 top panels. In this case, normal emission is possible because an x‐oriented dipole launches SPPs in the ± x directions that are of opposite phase (as for grating in‐coupling at normal incidence, *cf*. Figure 2a,b).

**FIGURE 8:**
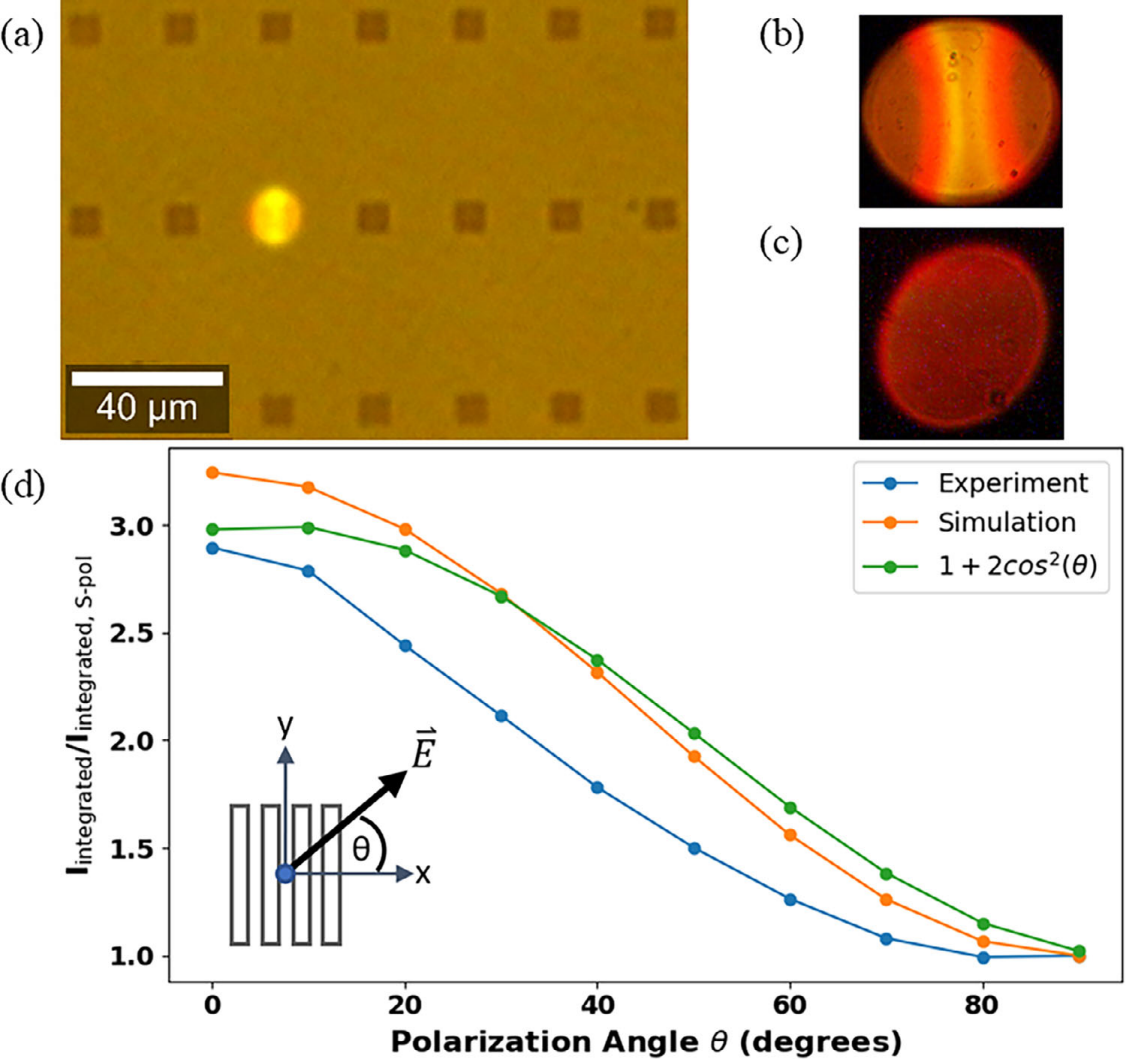
(a–c) Visible camera image of a luminescing grating in *N*_2_ collected with (a) commercial Raman microscope (Witec a300), and (b,c) via the inspection arm of the setup of Figure 1a. (d) Polarization of the PL emission from measurements and simulation (FDTD) compared with Malus's law. The polarization angle of the PL is relative to the grating ridges, as defined in the inset, i.e., θ = 0° implies p‐polarized PL, whereas θ = 90° implies s‐polarized PL.

Figure 8a shows a microscope image captured by a visible camera of a grating in N_2_ luminescing after 30 min of CW laser pumping (image captured by a commercial Raman microscope). Figure 8b,c shows images of two different gratings, luminescing after 30 min of CW pump exposure in N_2_ captured using a CMOS visible color microscope camera in the setup of Figure 1a. These images corroborate the modeling of Figure 7 in that the aggregate emission is primarily directed normal to the grating and into the first‐order diffraction lobes.

Figure 8d gives the polarization dependence of the PL emission captured in Figure 8c, measured using the setup of Figure 1a, but with a film polarizer added before the PL emission is coupled to the multimode fiber and the spectrometer. Counts are summed over the entire emission spectrum for each angle of the polarizer, then normalized to the case where the polarizer transmission axis is aligned to pass *s*‐polarized light relative to the grating. The polarization extinction ratio of the emitted PL is directly observed to be approximately 3:1 (5 dB).

Figure 8d also plots the polarization dependence of the far fields computed using the FDTD method. This result was obtained from the vectorial electric far fields radiated by the structure for our set of dipoles placed at the locations illustrated in Figure 4a and oscillating along the 3 axes (x, y, z). The vectorial far field produced by one dipole source was computed, passed numerically through a polarizer aligned at a specific angle relative to the grating, and the resulting associated intensity was integrated over the far‐field plane. The integrated intensity thus obtained for each dipole was then summed to represent total emission through the polarizer from the set. This calculation was repeated for each angle of the polarizer, and the results normalized to the *s*‐polarized case. A good agreement between measured and simulated results (and with the offset Malus's law) is obtained, as observed from Figure 8d.

The results in Figures 7 and 8 show that a set of randomly aligned and positioned incoherent emitters (carbon dots in this case) on a plasmonic metal grating produces light in the far field that exhibits characteristics of coherence such as directional emission in prescribed directions at specific wavelengths and with a specific polarization, as was previously shown for thermal emission [17].

## 3 Conclusions

In this work, we have demonstrated both experimentally and theoretically how plasmonic gratings significantly influence the formation and emission characteristics of nearby emitters. The role of surface plasmons in our study is four‐fold – first, pump plasmons catalyze a carbonaceous seed layer into photoluminescent carbon dots; second, pump plasmons excite the carbon dots; third, surface plasmons receive Stokes‐shifted quanta of energy emitted by decay of excited carbon dots; and fourth, surface plasmons scatter from the grating into specific free‐space radiation channels. Surface plasmons mediate the luminescence of the carbon dots, significantly altering their spontaneous emission lifetime and radiation characteristics into free space.

Incoherent, randomly positioned, and randomly aligned carbon dots, created in situ on a silver grating, were shown to produce polarized photoluminescence in prescribed directions and at specific wavelengths, which are properties normally associated with coherent light sources. Specifically, photoluminescence emerges preferentially along the grating normal and in the ± 1 diffraction directions, at wavelengths in the range of 560–700 nm (depending on the sample) when pumped at 532 nm, and with a *p*‐polarization exhibiting a 3:1 polarization extinction ratio. These coherence properties emerge because the emitters preferentially decay onto SPPs supported by the grating, which diffract therefrom with coherent characteristics. The photoluminescence lifetime of the carbon dots on gratings was measured to be in the range of 30–40 ps, which is about 70× shorter than the lifetime of carbon dots in free space. This is attributed to enhanced spontaneous emission into SPPs (due to enhanced near‐fields), for which a Purcell factor of about 70 is deduced. Our theory and modeling show strong agreement with experimental results, confirming the critical role of SPPs on a grating in shaping emission dynamics. These findings provide a deeper understanding of interactions between incoherent emitters and SPP modes, particularly carbon dots on silver gratings. The ability to localize and form carbon dots in situ on nanostructured surfaces, then control and enhance their emission characteristics with significantly shortened lifetimes, opens new possibilities for biosensing, inexpensive high‐speed light sources, and high‐brightness quantum optics.

## 4 Methods

### 4.1 Fabrication of Samples

A Si substrate was cleaned in ultrasonic baths of acetone, IPA, and DI water, followed by baking on a hot plate at 100°C for 10 min. A 100 nm thick Ag layer was deposited on a 10 nm thick Ti adhesion layer by electron‐beam evaporation (Angstrom Engineering NEXDEP) at rates of 5 and 1 Å/s, respectively. A positive photoresist layer (950 PMMA A2, Kayaku Advanced Materials) was spin‐coated at 3000 rpm for 1 min on the Ag layer, followed by baking on a hot plate at 180°C for 5 min.

A grating structure was formed by a helium ion beam lithography process described elsewhere [39], using a Zeiss Orion NanoFab helium ion microscope (HIM), equipped with Fibics Atlas engine (layout and exposure controlled using the Fibics Nanopatterning and Visualization Engine). Gratings were patterned into the PMMA layer using a helium ion beam current of 0.3 pA, at a landing energy of 25 kV, following a dot‐dose exposure pattern of step size x = y = 2 nm and dwell time of 0.5 µs, delivering a dose of 3.8 µC/cm^2^. Gratings with a 50/50 duty cycle covering an area of 30 × 30 µm^2^ were defined with a pitch of Λ = 500 nm. PMMA was developed in a 1:3 mixture of methyl isobutyl ketone (MIBK) and isopropanol (IPA) for 2 min at 20°C, followed by a 30 s IPA bath.

A 25 nm (target thickness) thick Ag layer was then deposited at a rate of 1 Å/s by thermal evaporation (Angstrom Engineering NEXDEP), at a working pressure at 5 × 10^−7^ Torr, to form the grating ridges. The sample was then submerged in acetone for 24 h at room‐temperature to lift off the remaining PMMA, thereby defining Ag grating ridges.

### 4.2 Helium Ion Microscopy (HIM)

High‐resolution HIM imaging was carried out on a Zeiss Orion NanoFab HIM. The top‐down image appearing in the inset to Figure 1a was acquired using a gas‐field ion source field of view (GFIS‐FOV) of 2 µm, a beam current of 1.33 pA, and a dwell time of 1 µs (1024 × 1024 pixels).

### 4.3 Atomic Force Microscopy (AFM)

AFM topographical scans of the Ag gratings were obtained in tapping mode using a Park Systems NX10 AFM equipped with a Tap300Al‐G tip to characterize surface morphology. The measured height and roughness (R_q_) of the fabricated gratings are 25 ± 1 and 2.6 ± 0.2 nm, respectively.

### 4.4 SERS Spectra

Surface enhanced Raman scattering (SERS) spectra were acquired using a custom setup as sketched in Figure 1a. The setup incorporates a custom‐machined gas chamber to confine gas flows of CO_2_ or N_2_ at ∼1.95 atm pressure. The excitation laser was a *p*‐polarized continuous wave (CW) laser at λ = 532 nm adjusted to produce 10 mW on the incident power on the grating. The laser beam was delivered to the grating via a dichroic mirror and a 20 × objective (Thorlabs RMS20X‐PF). The emission was collected with the same objective, passing through the dichroic mirror and two 6 OD notch filters (for a total of 12 OD at the notch) to attenuate the reflected pump beam. The emission was then focused into a multimode fiber using another objective and redirected to an electron‐multiplying intensified CCD (emICCD) camera connected to a spectrometer (Princeton Instruments). The setup also incorporates a beam splitter with imaging optics and a CMOS visible color microscope camera (UCMOS03100KPA) to visualize the sample and facilitating beam alignment and to capture images of the emitted PL. Spectra were compared against a commercial Raman microscope (WiTec a300 Raman microscope) for consistency (*cf*. Figure 2c).

### 4.5 Finite Element Method (FEM) Modeling

The interaction of the laser pump with the grating, as summarized in Figure 2a,b, was modeled using the FEM implemented in a commercial package (Comsol). A single unit cell of the grating was modeled in 2 dimensions with periodic boundary conditions applied laterally and absorbing boundary conditions applied along the top and bottom walls. A linearly polarized plane wave was launched onto the grating at a polarization angle of interest, and the reflectance, R, was computed in the far field above the grating. The absorptance plotted in Figure 2b was taken as 1 ‐ R. The material parameters for Ag in the modeling tool were used without modification.

### 4.6 Finite Difference Time Domain (FDTD) Modeling

The lifetime computations of Figure 4, the radiation plots of Figure 7, and the computed polarization plot of Figure 8 were obtained using the FDTD method implemented in a commercial package (Lumerical). A finite grating 2 µm long in the y‐direction and comprising 9 periods was modeled in a 3D volume bounded by perfectly matched layers to avoid unwanted field reflections from the terminating walls. In each numerical calculation, a single dipole oscillating along the x, y, or z axes was placed at one of the locations identified by the orange balls in Figure 4a and the table inset into Figure 4a. The locations are chosen to sample the entire central unit cell of the grating while maintaining the required computational resources at a reasonable level. The average decay rate of emitters close to the grating structure is then found by averaging over the decay rates of all dipole orientations at positions sampling the entire central unit cell. Locations highlighted by red dashed contours around the orange balls are mirrored locations corresponding to positions 2 to 6, and produce the same decay rate as their mirrored counterparts. Thus, an independent calculation was not performed for these locations, but their response was included in the decay rate averaging. This approach purports to model an ensemble of dipoles oriented randomly and located randomly over the grating surface. Further details on these computations and validation of our approach using the FDTD can be found in Section S.1.

### 4.7 Lifetime Measurements via Time‐Correlated Single Photon Counting (TCSPC)

The optical setup used to measure the emitter lifetime is shown in Figure 5a. It uses a supercontinuum source (NKT SuperK Extreme) as the pump, with a tunable acousto‐optic filter and a bandpass (line) filter of central wavelength λ = 532 nm, to deliver a ∼75 ps pulsed beam at a repetition rate of ∼78 MHz to the sample placed inside the gas chamber containing N_2_ or CO_2_ at a pressure of ∼1.95 atm. The beam was expanded to fill the aperture of the focusing objective for the best focusing resolution. The emission collected from the sample using the same objective was redirected to the collection arm by a dichroic mirror (Thorlabs DMLP550R). The pump signal reflected by the sample was attenuated by the dichroic mirror and two narrow bandstop (notch) filters, each of OD 6 at λ = 532 nm (for a total OD of 12) placed in the collection path. The collected emission was either coupled to a multimode fiber, which was redirected to a spectrograph and an emICCD camera to measure the emission spectrum (as in Figure 1a), or was focused into a single photon avalanche diode (SPAD) (MPD PDM series) connected to a TCSPC board (PicoQuant TimeHarp 260) to measure the emission lifetime. To measure the emission lifetime at λ = 633 nm A bandpass line filter was added in the collection arm right before the notch filters to only pass the emission component at λ = 633 nm.

To measure the IRF, a sample of zero lifetime consisting of a bare silver surface near a grating of interest (Figure 5b), or a bare silicon surface (Figure 6a), was placed in the setup and the lifetime measured. The only difference in the setup between the IRF measurement and the lifetime measurement of a sample was exchanging the samples in the chamber and replacing neutral density (ND) filters with the notch filters to block the pump. The decay curves measured as the setup IRF are shown in Figures 5b and 6a, and possess a Gaussian peak of temporal width ∼100 ps followed by an exponential tail with time delay of ∼330 ps due to carrier diffusion in the SPAD [34, 35]. The measurements in Figure 5b were obtained using a multimode fiber to redirect the emission onto the SPAD, while in Figure 6 all the curves were collected by free space coupling the emission into the SPAD to avoid pulse dispersion in the multimode fiber. These IRF functions are taken as the time‐discretized form of P(t) in Equation (7).

## Funding

Financial support provided by the Natural Sciences and Engineering Research Council of Canada, the Canada Foundation for Innovation, and the Canada Research Chairs program is gratefully acknowledged.

## Conflicts of Interest

The authors declare no conflict of interest.

## Supporting information




**Supporting File**: advs74750‐sup‐0001‐SuppMat.docx.

## Data Availability

The data that support the findings of this study are available from the corresponding author upon reasonable request.
