# Supplementary material for: Surface Plasmon Enhanced Photoluminescence of Carbon Dots Formed In Situ on Silver Gratings
Source: Adv Sci (Weinh). 2026 Mar 9;13(27):e23200. doi: 10.1002/advs.202523200 (PMC13170259; doi:10.1002/advs.202523200)
Supplement: Supplementary file 1 — Supporting File: advs74750‐sup‐0001‐SuppMat.docx. [file ADVS-13-e23200-s001.docx]

**Surface plasmon enhanced photoluminescence of carbon dots formed *in situ* on silver gratings**

Maryam Sadat Amiri Naeini,^1,2^ Jaspreet Walia,^2,3,†^ Luis-Angel Mayoral-Astorga,^2^ Hyung Woo Choi,^2,3^ Arnaud Weck,^1,2,4^ and Pierre Berini^1,2,3,*^

^1^Department of Physics, University of Ottawa, Ottawa, Ontario, Canada

^2^Nexus for Quantum Technologies Institute, University of Ottawa, Ottawa, Ontario, Canada

^3^School of Electrical Engineering and Computer Science, University of Ottawa, Ottawa, Ontario, Canada

^4^Department of Mechanical Engineering, University of Ottawa, Ottawa, Ontario, Canada

*Corresponding Author
^†^Presently with the Bank of Canada

**S.1 Theory of luminescent emitters near metal surfaces**

In the main text the lifetime of emitters close to a silver grating was studied. For such an investigation, it is educational to study emission near a metal surface. In a classical approach, emitters are considered as dipole oscillators, the spontaneous emission of which is back-reflected towards the emitters by the surface, driving them more strongly or weakly depending on the phase difference between the original emission and the reflected wave at the emitter’s location [1] [2]. As a result, the influence of a metal layer on the emission properties depends on the distance (h) between the emitter and the surface.

At distances of the order of $\lambda_{\mathrm{emission}}$ or slightly larger, the emitter couples to photons that can also be reflected from the surface. As a result, the lifetime as a function of perpendicular distance from the surface (τ(h)), possesses an oscillatory behavior due to constructive or destructive interference between emission and its reflection [2], as shown experimentally and theoretically [3] [4].

At distances larger than atomic dimensions (h > 5 nm) but smaller than $\lambda_{\mathrm{emission}}$, the emission is strongly coupled to the SPP modes supported by the metal, which then can be converted to free space photons by a grating coupler [2]. At very small distances, nonradiative decay channels into the metal, such as interband absorption (electron-hole pair generation), dipole-dipole interaction, and electron scattering, will dominate the process [2].

A theoretical model suggested in [4], considers the emitter as a point dipole and expands its field into a sum of plane waves of different wavevectors in the interface plane. By calculating the reflection coefficients for vertical and horizontal dipole orientations, and integrating over the reflected and original dipole fields, the spontaneous emission rate and dipole lifetime can be determined. Since the model uses the complex dielectric permittivity of the metal, it naturally includes the excitation of SPP modes at intermediate distances.

The normalized decay rate of a dipole located at a distance h from a metallic mirror is written [1] [5]:

$$\begin{aligned} \hat{\gamma}\left( h \right)=\hat{\gamma}_{\mathrm{nr}}^{0}+\eta_{0}\int_{0}^{\infty} \hat{P}\left( u,h \right)\mathrm{du}\#\left( S1 \right) \end{aligned}$$

where $\hat{\gamma}_{\mathrm{nr}}^{0}=1-\eta_{0}$ and $\eta_{0}$ are the normalized nonradiative decay rate and intrinsic quantum efficiency of the dipole respectively. $u={k_{\parallel}}/k$ is the wavenumber in the plane of the metal, normalized to the far field wavenumber. $\hat{P}\left( u,h \right)$ is the power dissipation density of the dipole as a function of $u$ and $h$, normalized to the far field dissipated power. $\hat{P}\left( u,h \right)$ is averaged over the power dissipation of all possible dipole orientations, resulting in an isotropic power dissipation:

$$\begin{aligned} \hat{P}\left( u,h \right)=\frac{2}{3}\hat{P}_{\parallel}\left( u,h \right)+\frac{1}{3}\hat{P}_{\perp}(u,h)\#\left( S2 \right) \end{aligned}$$

The normalized power dissipation components for the perpendicular ($\hat{P}_{\perp})$ and parallel ($\hat{P}_{\parallel}$) dipole orientations can be calculated as:

$$\begin{aligned} \hat{P}_{\perp}\left( u,h \right)=Real\left\{ \frac{3}{2}\frac{u^{3}}{\sqrt{1-u^{2}}} \left[ 1+r_{p}e^{i2k_{\perp}h} \right] \right\} \\ \hat{P}_{\parallel}\left( u,h \right)=Real\left\{ \frac{3}{4}\frac{u}{\sqrt{1-u^{2}}} \left[ \left( 1+r_{s}e^{i2k_{\perp}h} \right)+\left( 1-u^{2} \right)\left( 1-r_{p}e^{i2k_{\perp}h} \right) \right] \right\}\#\left( S3 \right) \end{aligned}$$

where $r_{p}$ and $r_{s}$ are Fresnel reflection coefficients of a multilayer structure including the metallic mirror, the material in which the dipoles reside at distance $h$ from the metal surface, and any layer above that, for waves polarized parallel (p) and perpendicular (s) to the interface, respectively. This model can be simply used for a two-layer environment (two semi-infinite media like air and silver) with $r_{p}$ and $r_{s}$ calculated to represent the Fresnel coefficient considering the interface of interest.

So, the lifetime of an isotropically oriented dipole above a metal surface can be written as a function of distance from the surface:

$$\begin{aligned} \tau\left( h \right)=\hat{\gamma}\left( h \right)^{-1}\tau_{0}\#\left( S4 \right) \end{aligned}$$

with $\tau_{0}$ being the intrinsic lifetime of the molecule.

Figure S1 shows results of calculations for a reference case consisting of a dipole in air close to a silver surface. The dipole is assumed to model R6G dye molecules which have an emission peak at $\lambda_{\mathrm{emission}}=560 nm$, a quantum efficiency of $\eta_{0}=0.9$ and an intrinsic lifetime of $\tau_{0}=3.9 ns$ at a concentration of 5 mM solved in ethanol [5].

Three different regimes on Fig. S1(a) can be identified. For small values of wavenumber (u), the emitters couple increasingly to far-field radiation with separation. The sharp peak around u = 1.045 shows coupling to the SPP mode, which has a wavevector larger than that of free-space, and as a result, is bound to the surface. This coupling increases as the emitter approaches the surface. At smaller distances, a broad feature at very large wavenumbers appears in the dissipated power trend which grows as the separation decreases. This represents coupling to non-radiative decay channels caused by any loss mechanism available in the metal (referred to as lossy surface waves in [1]).


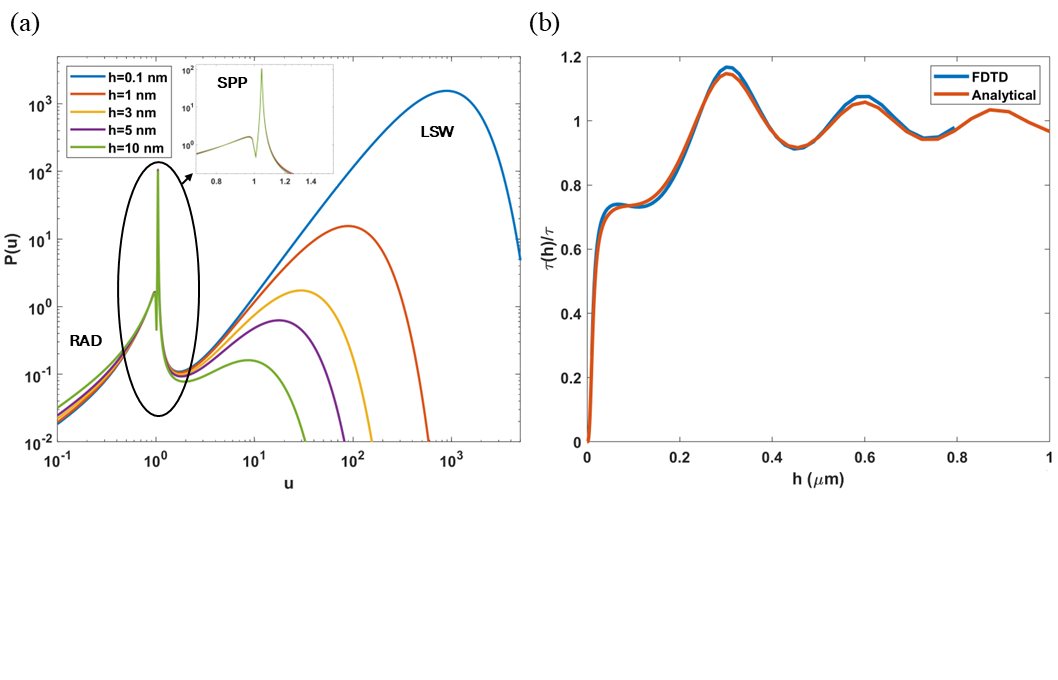


Figure S1. (a) Normalized power dissipation density *vs*. normalised wavenumber u for isotropically oriented dipoles in air near a silver surface at different distances. (b) Normalized lifetime of the same dipoles *vs*. the distance h from the surface calculated analytically using Eq. S1 and using the FDTD method as explained in the main text.

Figure S1(b) shows the normalized lifetime of isotropically oriented dipoles in air and close to a silver surface calculated analytically following Eq. S1 and compared with the FDTD method as explained in main text. The lifetime of dipoles reaches small values and eventually zero, with nonradiative decay channels dominating, as the dipole approaches the metal surface. At intermediate distances below $\lambda_{\mathrm{emission}}=560 nm$, emission couples to SPP modes and enhancement in lifetime is observed compared to the dipole intrinsic lifetime. SPPs provide means for the emission to couple into free space channels through surface roughness. At distances of order of $\lambda_{\mathrm{emission}}=560 nm$ and above, the lifetime shows an oscillatory behaviour with respect to the dipole distance from the surface as discussed and due to constructive or destructive interference between dipole emitted fields with the reflected fields from the surface.

To confirm the lifetime results calculated by the FDTD method in the main text, a comparison is made with the theoretical formulation. The quantum efficiency of a dipole source applied in the FDTD method is limited to a value of $\eta_{0}=1$. Figure S1(b) compares the FDTD simulations of the normalised lifetime of dipoles near a silver surface as a function of its separation h, to the theoretical results computed for $\eta_{0}=0.9$. Good agreement is observed between the two cases, confirming the accuracy of the FDTD method for quantum efficiencies of $\eta_{0}=0.9$ to $\eta_{0}=1$.

**S.2 Lifetime measurements of R6G dye**

Figure S2(a) gives the measured emission spectra of R6G dye solved in ethanol at a ${10}^{-6}$ M concentration, and as a coating of dried solution on a silver surface. The spectrum of the dye in solution is as expected based on the manufacturer’s data (Exciton, Rhodamine 590). The spectrum of the dried dye solution on the silver surface, captured under the same measurement conditions, is weaker and red-shifted. Weaker emission is not surprising because the dye is not efficiently pumped as a layer on a mirror (*cf*. Section 2 of the main text), and emission on the surface is preferentially into bound SPPs propagating thereon. The red-shifting of the spectrum is attributable to the dried layer of dye, which as an organic molecule, has a higher index of refraction than the ethanol used to form the solution. Emission therefore occurs in a higher index medium, leading to a change in the Stokes shift and thus the spectrum of emission based on the Lippert-Mataga equation [6].


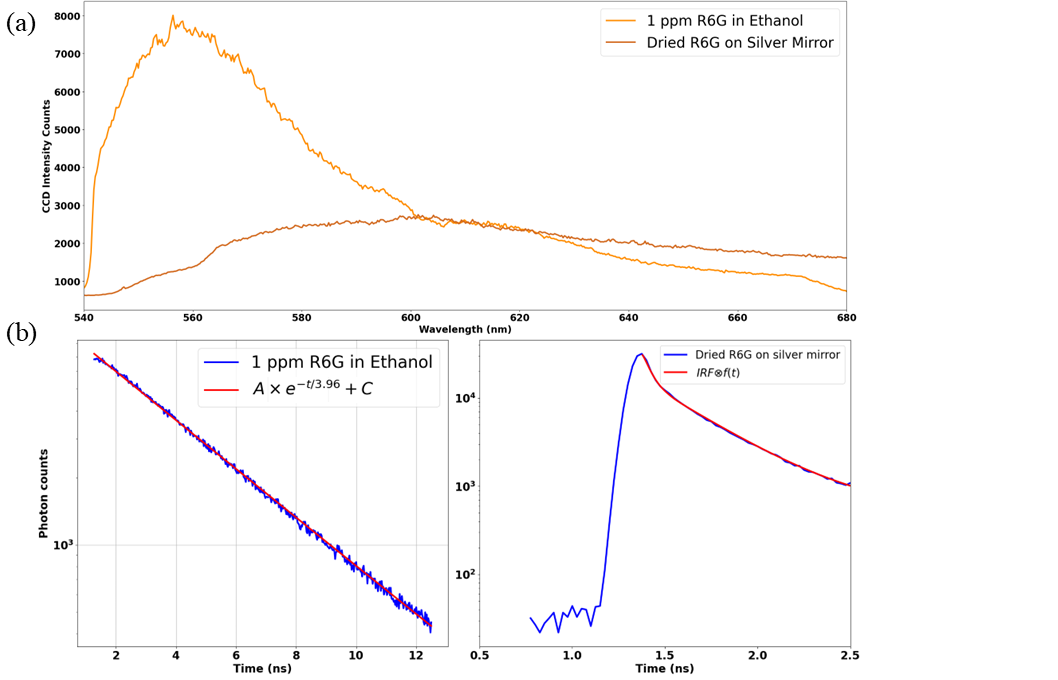


Figure S2. (a) Measured fluorescence emission spectra of the dye solution and of the dried layer on a silver surface. (b) Measured PL decay and extracted lifetime for R6G dye in a ${10}^{-6}$ M solution in ethanol, and as a dried layer of solution on a silver surface.

Fig. S2(b) gives PL decay of these spectra, measured using the optical setup shown in Fig. 5(a) of the main text. The best fit to the measured data for the dye solution consists of a single exponential decay function with an extracted lifetime of $\tau_{s}\simeq3.96 ns$ (Fig. S2(b), left panel), which agrees well with data available from the manufacturer (Exciton, Rhodamine 590). The decay of the dried solution on the silver mirror (Fig. S2(b), right panel) was modelled using the same double exponential function $f\left( t \right)$ as defined in the caption of Fig. 5(b) of the main text, from which a lifetime of $\tau_{m}\simeq30 ps$ was extracted for the dried dye.

The lifetime $\tau_{s}$ was measured in solution and is different than the intrinsic lifetime of the dye molecules in free space $\tau_{0}$. To determine the Purcell factor of the dye on the silver mirror, $\tau_{0}$ for the dye molecules must be calculated. The local density of optical states (LDOS) of a homogeneous dielectric volume is proportional to the square of the refractive index of the dielectric [7], and the decay rate is proportional to the LDOS following Fermi’s golden rule [1] [8]. As a result, the lifetime of dye molecules in free space decreases by a factor of $n_{e}^{2}$ with respect to the lifetime in ethanol. Using $n_{e}\simeq1.36$ for ethanol over $\lambda\simeq500-600 nm$, yields a Purcell factor of $\gamma=\left( \tau_{s}/n_{e}^{2} \right)/{\tau_{m}}\simeq70$, which is very close to the computed values for $\gamma_{\mathrm{Avg}}$ in Fig. 4(b) of the main text over the same wavelength range.

# References

| [1] | W. Barnes, "Fluorescence near interfaces: the role of photonic mode density," *Journal of Modern Optics,* vol. 45, no. 4, pp. 661-699, 1998. |
| --- | --- |
| [2] | G. Ford and W. Weber, "Electromagnetic interactions of molecules with metal surfaces," *Physics Reports,* vol. 113, no. 4, pp. 195-287, 1984. |
| [3] | K. Drexhage, "IV interaction of light with monomolecular dye layers," in *Progress in Optics*, vol. 12, Elsevier, 1974, pp. 163-232. |
| [4] | I. Prigogine and S. Rice, "Molecular Fluorescence and Energy Transfer Near Interfaces," in *Advances in chemical physics*, John Wiley & Sons, 2009, p. Vol. 37. |
| [5] | I. De Leon and P. Berini, "Modeling surface plasmon-polariton gain in planar metallic structures," *Optics Express,* vol. 17, no. 22, pp. 20191-20202, 2009. |
| [6] | B. Czaplińska, K. Malarz, A. Mrozek-Wilczkiewicz, A. Slodek, M. Korzec and R. Musiol, "Theoretical and experimental investigations of large stokes shift fluorophores based on a quinoline scaffold," *Molecules,* vol. 25, no. 11, p. 2488, 2020. |
| [7] | W. Barnes, S. Horsley and W. Vos, "Classical antennas, quantum emitters, and densities of optical states," *Journal of Optics,* vol. 22, no. 7, p. 073501, 2020. |
| [8] | E. Fermi, "Quantum theory of radiation. , 4(1), p.87.," *Reviews of Modern Physics,* vol. 4, no. 1, p. 87, 1932. |
